# Who Wants to Enhance Their Cognitive Abilities? Potential Predictors of the Acceptance of Cognitive Enhancement

**DOI:** 10.3390/jintelligence11060109

**Published:** 2023-06-01

**Authors:** Sandra Grinschgl, Anna-Lena Berdnik, Elisabeth Stehling, Gabriela Hofer, Aljoscha C. Neubauer

**Affiliations:** 1 Institute of Psychology, University of Graz, Universitätsplatz 2, 8020 Graz, Austria; gabriela.hofer@uni-graz.at (G.H.); aljoscha.neubauer@uni-graz.at (A.C.N.); 2 Institute of Psychology, University of Bern, Fabrikstrasse 8, 3012 Bern, Switzerland

**Keywords:** cognitive enhancement, intelligence, self-estimates, personality, Dark Triad, science-fiction

## Abstract

With advances in new technologies, the topic of cognitive enhancement has been at the center of public debate in recent years. Various enhancement methods (e.g., brain stimulation, smart drugs, or working memory training) promise improvements in one’s cognitive abilities such as intelligence and memory. Although these methods have been rather ineffective so far, they are largely available to the general public and can be applied individually. As applying enhancement might be accompanied by certain risks, it is important to understand which individuals seek to enhance themselves. For instance, individuals’ intelligence, personality, and interests might predict their willingness to get enhanced. Thus, in a preregistered study, we asked 257 participants about their acceptance of various enhancement methods and tested predictors thereof, such as participants’ psychometrically measured and self-estimated intelligence. While both measured and self-estimated intelligence as well as participants’ implicit beliefs about intelligence, did not predict participants’ acceptance of enhancement; a younger age, higher interest in science-fiction, and (partially) higher openness as well as lower conscientiousness did. Thus, certain interests and personality traits might contribute to the willingness to enhance one’s cognition. Finally, we discuss the need for replication and argue for testing other potential predictors of the acceptance of cognitive enhancement.

## 1. Introduction

In the 21st century, new powerful technologies, such as different artificial intelligence (AI) agents, have become omnipresent and the center of public debate. With the increasing fear of AI agents replacing humans, there are discussions about whether individuals should strive to enhance themselves. For instance, the philosophical movement *Transhumanism* proposes the broad enhancement of human characteristics such as cognitive abilities, personality, and moral values (e.g., Grassie and Hansell 2011; Ranisch and Sorgner 2014). This enhancement should help humans to overcome their natural limitations and to keep up with powerful technologies that are increasingly present in today’s world (see Ranisch and Sorgner 2014). In the present article, we focus on one of the most frequently discussed forms of enhancement—the enhancement of human cognitive abilities.

Not only in science but also among the general population, cognitive enhancement, such as increasing one’s intelligence or working memory capacity, has been a frequently debated topic for many years (see Pauen 2019). Thus, a lot of psychological and neuroscientific research investigated different methods to increase cognitive abilities, but—so far—effective methods for cognitive enhancement are lacking (Jaušovec and Pahor 2017). Nevertheless, multiple different (and partly new) technologies that promise an enhancement of cognition are available to the general public. Transhumanists especially promote the application of brain stimulation techniques, smart drugs, or gene editing for cognitive enhancement (e.g., Bostrom and Sandberg 2009). Importantly, only little is known about the characteristics of individuals who would use such enhancement methods to improve their cognition. Thus, in the present study, we investigated different predictors of the acceptance of multiple widely-discussed enhancement methods. More specifically, we tested whether individuals’ psychometrically measured intelligence, self-estimated intelligence, implicit theories about intelligence, personality (Big Five and Dark Triad traits), and specific interests (science-fiction hobbyism) as well as values (purity norms) predict their acceptance of cognitive enhancement (i.e., whether they would use such methods to enhance their cognition).

### 1.1. Overview of Cognitive Enhancement Methods

Cognitive enhancement^1^ describes the enhancement of different cognitive abilities in healthy individuals (Viertbauer and Kögerler 2019). Thus, it needs to be differentiated from compensatory enhancement, which is applied for therapeutic reasons when individuals have certain disabilities or illnesses and need to compensate for those (Birnbacher 2019). For the former (cognitive enhancement), transhumanists advertise different methods—most of which arise from the technological progress in the last decades. Some frequently discussed enhancement methods are pharmacological enhancement, current-based enhancement, genetic enhancement, and mind upload (e.g., Bostrom and Sandberg 2009; Loh 2020).

**Pharmacological enhancement** describes the intake of certain drugs (e.g., substances based on modafinil, amphetamine, or methylphenidate), not for their prescribed use but to enhance one’s central nervous system (see Repantis et al. 2010). While these drugs are usually used to treat disorders such as attention deficit hyperactivity disorder (Schifano et al. 2022), there seems to be an increase in their use for cognitive enhancement (Esposito et al. 2021; Maier et al. 2018). However, the effectiveness of so-called *smart drugs* for cognitive enhancement seems rather mixed (e.g., Daubner et al. 2021). Furthermore, their intake might be accompanied by certain risks, such as addiction (Massie et al. 2017; Sharif et al. 2021).

**Current-based enhancement** entails the application of different brainstimulation techniques, such as transcranial electric or deep brain stimulation. This enhancement method is used to increase cognitive abilities such as working memory (Jaušovec and Pahor 2017; Luber et al. 2007), but findings on the effectiveness of current-based enhancement are mixed so far. For instance, a meta-analysis by Simonsmeier et al. (2018) showed transcranial electric stimulation had stronger positive effects when applied during learning compared to test performance—but only for anodal and not cathodal stimulation and the effectiveness was dosage-specific. Thus, the effectiveness of current-based enhancement seems to depend on multiple characteristics of the applied stimulation. Due to the increasing availability of current-based enhancement methods, Santarnecchi et al. (2015) argued for guidelines and regulations to ensure users’ safety. This is especially important as it is unclear how the regular use of current-based enhancement might affect one’s brain (e.g., Shah-Basak and Hamilton 2017).

**Genetic enhancement** might become an especially powerful enhancement method. The possibility of altering human genes allows for the optimization of body and cognition—even before a child is born. Thus, already within the fetus, supposedly undesired characteristics can be modified (which is sometimes also called prenatal and perinatal enhancement; Bostrom and Sandberg 2009). Research has already identified genes relevant to intelligence (Plomin and von Stumm 2018). Thus, in the future, those might be modified to enhance cognition. Such gene editing poses important ethical questions, such as who can decide when modifying the genes of a fetus (see e.g., Bostrom and Sandberg 2009).

**Mind upload** describes a rather futuristic cognitive enhancement method that is commonly part of science-fiction literature and movies. It refers to the possibility of uploading one’s personality, intelligence, memories, and other characteristics onto an external hard drive so that one can live digitally and forever—independent of one’s body (e.g., Laakasuo et al. 2018). Although this idea seems unrealistic currently, it is frequently debated by transhumanists, and there are even ongoing projects within and outside the European Union that are investigating the potential digitalization of the brain (e.g., the Neurotwin project^2^).

The four aforementioned methods do not require any training or other active participation from the user to (supposedly) enhance cognition. Thus, without any effort, these methods promise to increase cognition—just by, for instance, taking drugs or applying brain stimulation. We refer to these four methods as passive enhancement methods. However, other techniques that involve the active participation of the user to achieve the desired effect have also been discussed. Often-named examples of such active enhancement methods are working memory training, game-based enhancement, neurofeedback training, and brain-machine interfaces (e.g., Bostrom and Sandberg 2009; Jaušovec and Pahor 2017).

**Working memory training** might enhance individuals’ working memory capacity and potentially also their intelligence. For instance, in a study by Jaeggi et al. (2008), working memory training was shown to improve working memory capacity (near-transfer effect) as well as fluid intelligence (far-transfer effect). However, replication studies failed to reproduce the far-transfer effect (Melby-Lervåg and Hulme 2013; Shipstead et al. 2012). Thus, it is questionable whether completing working memory training enhances individuals’ cognition beyond the trained task. Nevertheless, many tools that promise a broad cognitive enhancement via working memory training are available to the general public. For instance, on 19 January 2023, the Google Play Store offered more than 30 apps for “working memory training”.

**Game-based enhancement** is another form of active enhancement that involves improving one’s cognition by playing video games. Studies have shown positive effects of gaming on cognitive abilities (Green and Bavelier 2003; see also Oei and Patterson 2013). Additionally, a study by Ninaus et al. (2015) showed a positive effect of gaming elements on performance and efficiency in working memory tasks. Nevertheless, it is unclear whether the positive effects of playing games on cognition are stable over time or can be transferred to settings outside the gaming context (see Ninaus et al. 2015; Oei and Patterson 2013).

**Neurofeedback training** is another non-invasive method that aims at training individuals to deliberately control their brain activity. Individuals get visual, auditory, or haptic feedback on their brain activity with, for instance, electroencephalography or functional magnetic resonance imaging. The goal is that individuals learn to increase and decrease certain brain activities (e.g., the sensorimotor rhythm; Kober et al. 2018), which, in return, might foster motor activity, affect, and cognition (Enriquez-Geppert et al. 2017; Kober and Wood 2020). Neurofeedback training is used in clinical settings to, for instance, treat patients after having a stroke (e.g., Kober et al. 2015). For healthy individuals, the first studies also suggest positive effects of neurofeedback training such as on working memory (Kober et al. 2015) or attention (Gruzelier 2014). However, additional research is necessary to systematically test the effectiveness of neurofeedback training as an enhancement method (see Kober and Wood 2020).

**Brain-machine interfaces**^3^ describe the connection between a brain and a computer through, for instance, brain implants that allow the control of computers. Through brain-machine interfaces, machines can be controlled by the brain, without manually interacting with them (e.g., Silva 2018). In addition to receiving a brain implant, oftentimes intensive training is required to achieve a connection between the brain and the computer (Silva 2018). Currently, scientists test whether brain-machine interfaces can be used to transfer words (Moses et al. 2019) or to move robotic extremities by the power of thought (for a review article on this behalf see Dominijanni et al. 2021). A well-known institution working on brain-machine interfaces is Neurolink, led by Elon Musk. Their goal is to use brain-machine interfaces not only for humans with neurological disorders but also to enable a connection between (healthy) humans and artificial intelligence. However, the use of brain-machine interfaces is accompanied by multiple challenges such as the *neural resources allocation problem* which describes “the channeling of motor commands and sensory information to and from the augmentative device without hindering the motor control of biological limbs” (Dominijanni et al. 2021, p. 851).

These four active enhancement methods require the user to be actively involved in the process to (supposedly) enhance their cognition by, for instance, conducting multiple training sessions. These methods, thus, require more effort than passive enhancement methods. Notably, we derived these two groups of enhancement methods (passive and active) from the literature. However, we also conducted an exploratory factor analysis to test whether we can confirm this structure empirically (see results).

### 1.2. Predictors of the Acceptance of Cognitive Enhancement

The broad application of cognitive enhancement might be accompanied by many societal changes (see Neubauer 2021). For instance, access to enhancement could increase social inequality if wealthy people could afford enhancement more easily than poor people. Furthermore, if enhancement proves to be effective, there might be pressure for individuals to get enhancement by, for instance, their employers. Additionally, if everyone can become smarter, the question arises whether a majority will become academics, leading to a lack of people in blue-collar jobs (see Neubauer 2021). Due to the (ethical) challenges enhancement might raise for society but also due to the potential risks for individuals (e.g., long-term damages or addiction), it is highly important to investigate who wants to enhance themselves while these methods are still being developed. If the characteristics of individuals who want to get enhanced are known, they can be specifically targeted to sensitize them to the correct application of different enhancement methods and inform them about their potential side effects.

So far, psychological research and, especially individual-differences research, investigating factors related to cognitive enhancement is rather absent (see Neubauer 2021). Only a few studies investigated individuals’ assumptions about and acceptance of enhancement up to now (e.g., Breivik et al. 2022; Grinschgl et al. 2022; Mayor et al. 2020; Laakasuo et al. 2018; Schönthaler et al. 2022). For instance, Laakasuo et al. (2018; see also 2021) tested—among other factors—how personality traits, science-fiction hobbyism, and purity norms are related to individuals’ feelings and reactions toward mind upload. Purity norms include values such as pureness, naturalness, and decency. While a higher interest in science-fiction was related to a higher approval of mind upload, stronger purity norms were associated with less approval. Thus, exposure to and familiarity with futuristic ideas such as in science-fiction content seems relevant when it comes to the acceptance of enhancement. Furthermore, the purity of one’s mind and actions might drive the acceptance of enhancement. In the same study, Laakasuo et al. (2018) observed no significant relationships between personality factors and acceptance of mind upload.

Grinschgl et al. (2022) investigated individuals’ assumptions about four different passive enhancement methods. Individuals’ openness was related to more negative assumptions about most enhancement methods. However, the observed effects were only small and no other consistent findings with variables such as basic human values were observed. In another study, Schönthaler et al. (2022) tested the Big Five traits and sub-facets as well as the Dark Triad traits and basic human values as predictors of the acceptance of enhancement. While extraversion, neuroticism, and openness were not related to the acceptance of enhancement, lower agreeableness and conscientiousness were related to more acceptance of cognitive enhancement. This supports the idea that the traits of agreeableness and conscientiousness are related to avoiding risky behaviors (see Schönthaler et al. 2022). In addition, conscientious individuals might view enhancement (and related performance-gains) as unfair and not authentic. With regard to basic human values, lower self-transcendence values but higher self-enhancement values were related to more acceptance of enhancement. Moreover, Schönthaler et al. (2022) observed that the Dark Triad traits (Machiavellianism, psychopathy, and grandiose narcissism) and vulnerable narcissism were positively related to the acceptance of enhancement. Thus, individuals high on Dark Triad traits might be associated with showing ethically questionable behaviors (see e.g., Harrison et al. 2018) such as applying enhancement. In addition, those individuals might view enhancement as a promising strategy to fulfill their self-centered goals (see Schönthaler et al. 2022). Yet, in multiple regression, these dark traits showed no incremental validity beyond the predicting sub-facets of agreeableness and conscientiousness as well as values.

### 1.3. The Present Study

Previous studies showed that certain personality traits, values, and interests are related to the acceptance of cognitive enhancement—but only to a rather small degree. As cognitive enhancement methods are specifically targeting the enhancement of core cognitive abilities, such as working memory and intelligence, individual differences in these abilities might contribute to predicting the acceptance of enhancement. Similarly, research showed that intelligence is related to, for instance, illegal drug use (White and Batty 2012). These authors observed a positive association between childhood IQ and later drug use, which might be explained by a high need for stimulation in intelligent individuals. This suggests that intelligence might also play a role when it comes to related behaviors such as cognitive enhancement. To our best knowledge, however, no study has tested psychometrically measured and self-estimated intelligence as potential predictors of cognitive enhancement. Thus, the goal of the present study was to investigate multiple different predictors of enhancement, such as intelligence, different personality traits, and specific interests as well as values, and to test how they might together explain the acceptance of cognitive enhancement.

For the potential relationship between intelligence and the acceptance of enhancement, two conflicting hypotheses can be derived: the *rich-get-richer hypothesis* and the *compensation hypothesis*.

The rich-get-richer hypothesis suggests that individuals who already have high cognitive abilities (i.e., high intelligence) want to increase their abilities even further and thus might be willing to enhance themselves. This hypothesis is related to the so-called *Matthew effect* (Merton 1968), which suggests that it is easier for individuals high in some traits to increase those. For individuals low in some traits, it is harder to increase them (see also Neubauer 2021). Thus, more intelligent individuals might have higher chances of further increasing their intelligence than less intelligent individuals. Due to this assumption, more intelligent individuals might also be more willing to enhance themselves. If this rich-get-richer hypothesis applies to the acceptance of cognitive enhancement, we might observe a positive relationship between measured and self-estimated intelligence and the acceptance of passive and active enhancement methods.

The compensation hypothesis suggests that less intelligent individuals might be more drawn toward enhancement to compensate for their lack of cognitive abilities. This effect is based on the so-called *reverse Matthew effect* suggesting that less intelligent individuals profit more from enhancement, which ultimately might lead to the closure of the gap between more and less intelligent individuals (see Neubauer 2021; Schroeders et al. 2016). Based on the compensation hypothesis, we might observe a negative relationship between individuals’ measured and self-estimated intelligence and their acceptance of passive and active enhancement methods.

As there does not appear to be any empirical evidence that one of these two hypotheses might be more likely when it comes to the acceptance of cognitive enhancement, we decided to investigate the relationship between measured/self-estimated intelligence and the acceptance of enhancement with an open research question. Thus, these main research questions were preregistered as follows^4^:

*RQ1: Are there significant correlations between a person’s measured intelligence and the acceptance of “active” or “passive” enhancement methods?*

*RQ2: Are there significant correlations between a person’s self-estimated intelligence and the acceptance of “active” or “passive” enhancement methods?*

It should be noted, that we might not observe the same outcomes for both measured and self-estimated intelligence, as a series of previous studies showed that the two variables are only moderately correlated (e.g., see Freund and Kasten 2012; Neubauer and Hofer 2021; Zell and Krizan 2014). Thus, individuals are not always aware of their intelligence and might base their decisions on inaccurate metacognitive beliefs (see Hofer et al. 2022; Neubauer and Hofer 2021). While self-estimated intelligence might be a predictor of the acceptance of enhancement (e.g., individuals estimating their intelligence higher might be more drawn towards enhancement), it is plausible that psychometrically measured intelligence might only play a small role.

In addition to measured and self-estimated intelligence, individuals’ beliefs (i.e., implicit theories) about intelligence might also predict the acceptance of cognitive enhancement. Dweck and Leggett (1988) described two opposing implicit theories of intelligence: Following the entity theory, individuals view intelligence as immutable and, thus, as a fixed trait. In contrast, the incremental theory suggests that intelligence can be improved with enough effort. Believing in this latter theory was previously associated with putting more effort into achieving learning goals (Blackwell et al. 2007). In the present study, we were interested in testing whether those implicit beliefs about intelligence predict the willingness to get enhanced. If individuals believe that intelligence can be changed by certain means (incremental theory), they might be more accepting of cognitive enhancement. However, as we know of no previous study investigating this relationship, we tested it with an open research question:

*RQ3: Are there significant correlations between a person’s implicit theories of intelligence and acceptance of “active” or “passive” enhancement methods?*

Importantly, implicit theories of intelligence might show similar or different associations to passive and active enhancement methods. Arguably, individuals more drawn towards the incremental theory might be more willing to put effort into improving their cognition (e.g., see Blackwell et al. 2007) and, thus, show a higher acceptance of active but not passive enhancement.

As already mentioned, previous research on the acceptance of cognitive enhancement mainly focused on personality as a predictor. In this study, we also assessed participants’ Big Five personality and their Dark Triad traits (complemented by vulnerable narcissism) to test their incremental validity beyond measured/self-estimated intelligence and implicit theories of intelligence. With regard to the Big Five traits, agreeableness and conscientiousness might be related to the acceptance of enhancement. As suggested by Schönthaler et al. (2022), these traits might account for avoiding risky behaviors leading to less acceptance of enhancement. Furthermore, agreeableness is usually associated with following group norms and conscientious individuals might view enhancement as an unfair performance advantage. On the other hand, extraversion, openness, and neuroticism do not seem relevant when it comes to the acceptance of cognitive enhancement—at least not when enhancement is framed in a rather generic context without inducing performance-challenging situations (see Schönthaler et al. 2022). Thus, we derived the following (preregistered) hypothesis regarding the Big Five traits (for a full breakdown of these and all other hypotheses see Table 1):

**H1.**  *We expect significant negative correlations between the acceptance of “active” or “passive” enhancement methods and the Big Five traits of agreeableness and conscientiousness and virtually no association with the Big Five traits of extraversion, openness, and neuroticism.*

Also following the previous study by Schönthaler et al. (2022), we considered the Dark Triad traits and vulnerable narcissism as predictors of the acceptance of cognitive enhancement. The possibility to improve one’s performance via enhancement might be promising for individuals high on dark traits to achieve their goals. In addition, they might not worry about the rather unethical aspects of enhancement and, thus, be more willing to apply it (see Schönthaler et al. 2022). We, therefore, derived the following (preregistered) hypothesis:

**H2.**  *We expect significant positive correlations between the acceptance of “active” or “passive” enhancement methods and the Dark Triad traits (Machiavellianism, psychopathy, grandiose narcissism) and vulnerable narcissism.*

In addition to intelligence and personality, specific interests and norms of individuals might predict their acceptance of cognitive enhancement. Following the study by Laakasuo et al. (2018), we tested science-fiction hobbyism and purity norms as potential predictors of enhancement. Familiarity with transhumanistic ideas—as often presented in science-fiction literature and movies—might be positively related to the acceptance of cognitive enhancement. On the other hand, the emphasis on the importance of one’s purity might be negatively associated with the acceptance of enhancement. We, thus, derived the following (preregistered) hypotheses:

**H3.**  *We expect a significant positive correlation between science-fiction hobbyism and the acceptance of “active” or “passive” enhancement methods.*

**H4.**  *We expect a significant negative correlation between purity norms and the acceptance of “active” or “passive” enhancement methods.*

As a final research question, we aimed at testing whether these factors explain variance in the acceptance of passive/active enhancement methods in hierarchical multiple regression models. We, thus, preregistered the following research question:

*RQ4: Are measured intelligence, self-estimated intelligence, and implicit theories of intelligence able to predict statistically significant variance in the acceptance of “active” or “passive” enhancement methods in addition to personality traits (Big Five, Dark Triad, vulnerable narcissism)?*

We additionally preregistered that we will also include science-fiction hobbyism and purity norms in these analyses if they significantly correlate with the acceptance of passive/active enhancement and our main variables (intelligence and personality measures) do not explain at least a moderate variance in the acceptance of enhancement (for details see https://osf.io/urwxt).

To summarize, in the present study, we investigated a set of factors that might account for individual differences in the acceptance of passive and/or active enhancement methods. We, therefore, tested whether individuals’ psychometrically measured and self-estimated intelligence as well as their implicit theories about intelligence are related to the acceptance of enhancement. Replicating previous studies (e.g., Laakasuo et al. 2018; Schönthaler et al. 2022), we also investigated individuals’ Big Five personality and Dark Triad traits as well as their science-fiction hobbyism and purity norms. Finally, in hierarchical multiple regression models, we aimed at testing predictors that contribute to explained variance in the acceptance of enhancement. Thus, our study includes a broad range of factors that might contribute to the acceptance of enhancement—an increasingly important topic as more and more enhancement methods become publicly available and known.

## 2. Methods

This study was preregistered via the Open Science Framework (https://osf.io/urwxt). All deviations from the preregistration are declared within this article. In addition, data and analysis scripts are openly available (https://osf.io/du39z/).

### 2.1. Participants

The study was conducted online using the survey platform Unipark in two parts (see study procedure). The participants were required to be between 18 and 64 years old^5^ and to speak German as their mother tongue or to have equivalent German language skills. We recruited participants via social media websites, flyers at the local university campus, announcements in university lectures, university mailing lists, and with the help of Probando—a local participant recruitment company. As an incentive for study participation, we offered the participants feedback in the form of their personality profiles. Furthermore, psychology students could earn course credits.

As our main research questions and hypotheses entail correlational analyses, we based our sample size considerations on a simulation study by Schönbrodt and Perugini (2013): According to their simulation, moderate correlations should stabilize at a sample size of 250 participants. Thus, we originally aimed for 300 participants to complete both survey parts. However, as data collection was more difficult than expected, we updated our preregistration to end data collection by 24 July 2022 even if 300 full data sets^6^ were not reached.

At the end of this data collection period, we had obtained full data sets of 263 participants. For this sample, our preregistered inclusion criteria (i.e., age range, German language level, and completion of both survey parts) were fulfilled. We unexpectedly had to exclude three participants who reported having used unauthorized help (e.g., a calculator) and three participants who did not properly answer the number series task (i.e., instead of providing the numeric solution as an answer, they provided a written explanation for the logic of the number series). Our final sample consisted of 257 participants (172 female, 85 male). Their mean age was 29.25 years (*SD* = 11.35; 18–64 years old). Most participants had completed the A-levels or already received a university degree (216 participants). Furthermore, the majority indicated being interested in technology (167 participants) and a third of participants indicated studying psychology (85 participants). All participants provided informed consent before study participation. The study was approved by the local ethics committee.

### 2.2. Materials

All materials were administered in their German version. For descriptive statistics of all variables, see Table 2.

**Acceptance of Enhancement.** Participants’ acceptance of enhancement was measured with an adapted and extended questionnaire based on the one used by Schönthaler et al. (2022). It includes short vignettes (4 to 7 sentences) about eight enhancement methods that can (theoretically) be differentiated into passive and active enhancement methods. Passive enhancement methods refer to the improvement of cognition by a one-time use of the enhancement method:

The *pharmacological enhancement* vignette describes the intake of a drug to increase cognition by increasing the activation of certain brain regions.

The *current-based enhancement* vignette describes the use of brain stimulation such as transcranial electrical stimulation to increase cognition.

The *genetic enhancement* vignette refers to genome design in order to influence genes that are relevant for cognition.

The *mind upload* vignette describes the future possibility of uploading one’s brain and thereby becoming immortal.

Active enhancement methods are described as being successful in improving cognition when being applied regularly with a certain effort:

The *working memory training* vignette refers to app-based training conducted for several weeks to substantially improve memory.

The *game-based enhancement* vignette describes a virtual game that aims at increasing neural growth and, thus, improving intelligence with regular use.

The *neurofeedback training* vignette describes a neuro-gym in which individuals can train the regulation of their brain activity and, therewith, improve cognition throughout multiple training sessions.

The *brain-machine interface* refers to getting an implanted brain chip and completing intense training to learn controlling machines by the power of thoughts.

After reading each vignette, the participants responded to three questions on a six-point Likert scale ranging from “1—strongly disagree” to “6—strongly agree”. First, they were asked whether they would use the respective enhancement method to increase their cognitive abilities. For active enhancement methods, this referred to regular use of the respective method. This question served as an indicator of individuals’ acceptance of the respective method. Second, participants were asked to rate whether they think it is likely that this enhancement method will exist in the future. Third, they were asked to indicate whether this enhancement method would be successful in enhancing their cognitive abilities.^7^ We piloted this adapted and extended questionnaire with nine participants to test the vignettes’ comprehensibility. We received small suggestions for improvement which we incorporated into the final questionnaire. The questionnaire—translated into English—can be accessed at https://osf.io/du39z/.

To measure participants’ acceptance of enhancement, we planned (and preregistered) to average scores across the four passive (as an indicator of acceptance of passive enhancement) and four active enhancement methods (as an indicator of acceptance of active enhancement). However, as preregistered, we first conducted an exploratory factor analysis to test the underlying structure of acceptance towards the eight enhancement methods. This factor analysis showed that the acceptance ratings of the four passive enhancement vignettes as well as the brain-machine interface vignette loaded onto one common factor, whereas the acceptance of the remaining three active enhancement vignettes loaded on a second factor (for a detailed description, see the results section). Thus, based on these results, we averaged the ratings of the following five vignettes for an indicator of acceptance of passive enhancement: pharmacological enhancement, current-based enhancement, genetic enhancement, mind upload, and brain-machine interface. As an indicator of active enhancement, we averaged the acceptance ratings of the remaining three vignettes: working memory training, game-based enhancement, and neurofeedback training. Internal consistency was good for both passive and active enhancement methods (see Table 2).

**Self-estimation Measures.** To measure participants’ self-estimated intelligence, we used single-item scales on which participants were required to estimate their intelligence quotient (IQ) in relation to the population (for previous use of these scales. see Hofer et al. 2022). Participants were presented with the normal distribution of intelligence in the population and needed to indicate their own IQ on a scale from 55 (“slightly impaired”) to 145 (“highly gifted”). Participants did so once for their general intelligence and also separately for their numerical, verbal, and spatial intelligence. For our main analyses, we used the first estimate, namely the participants’ estimate of their general intelligence.

In addition to these single-item scales, we also asked participants to estimate their numerical, verbal, and spatial intelligence on a multi-item questionnaire (e.g., Neubauer and Hofer 2021). This questionnaire included ten questions each for numerical and spatial intelligence as well as nine questions targeting verbal intelligence. Participants answered those questions on a five-point Likert scale ranging from “1—not true at all” to “5—exactly true”. A mean value for each subscale was computed (higher means indicate a higher self-estimation). We administered this multi-item questionnaire for exploratory purposes but did not consider it in our main analyses.

**Intelligence Measures.** To measure participants’ intelligence—and more specifically their numerical, verbal, and spatial intelligence—we administered subscales of the Intelligenz-Struktur-Analyse (ISA; Fay et al. 2001). To measure numerical intelligence, the task “number series” was used. We slightly increased the task difficulty by reducing the time limit from eleven to eight minutes as previous studies showed ceiling effects in this task (e.g., Hofer et al. 2022). We piloted this adapted task with eight participants and did not observe ceiling effects. Verbal intelligence was measured with the task “analogies” which had a time limit of seven minutes. The third intelligence domain—spatial intelligence—was measured with a figure assembling task that had a time limit of seven minutes. Each task included 20 questions and sum scores were calculated for each domain. Additionally, as our main variable of interest, a general intelligence score for each participant was computed by averaging the z-transformed sum scores of all three intelligence domains.

**Implicit Theories of Intelligence.** Whether a person perceives intelligence as fixed or malleable was assessed by the Implicit Theories of Intelligence Scale (ITIS; Troche and Kunz 2020). This scale contains eight items that participants answered on a six-point Likert scale ranging from “1—I agree strongly” to “6—I disagree completely”. Four items are directed toward the entity theory and four items toward the incremental theory. To assess a person’s implicit theory of intelligence, the four entity theory items were recoded (see Troche and Kunz 2020) and a mean value over all eight items was computed. Higher scores indicate that participants believe more in an entity theory of intelligence.

**Big Five Personality.** To assess the Big Five personality traits, we used the short version of the Big Five Inventory (BFI-K; Rammstedt and John 2005). The questionnaire contains 21 items that are rated on a five-point Likert scale ranging from “1—very inapplicable” to “5—very applicable”. As indicators of the five traits (agreeableness: 4 items; conscientiousness: 4 items; extraversion: 4 items; openness: 5 items; neuroticism: 4 items), mean values were computed for each trait using the corresponding items.

**Dark Triad Traits.** Participants’ dark personality was measured using the Dirty-Dozen questionnaire (Küfner et al. 2015). The questionnaire contains 12 items—four items for each Dark Triad trait (Machiavellianism, psychopathy, and grandiose narcissism). Participants were asked to rate these items on a nine-point Likert scale ranging from “1—not true at all” to “9—exactly true”. We computed mean values for each Dark Triad trait using the respective items.

**Vulnerable Narcissism.** To assess vulnerable narcissism, we used the Hypersensitive Narcissism Scale (HSNS; Hendin and Cheek 1997). The scale consists of ten items and a five-point Likert scale ranging from “1—strong disagreement” to “5—strong agreement”. A mean value was computed over all items to indicate vulnerable narcissism.

**Science-fiction Hobbyism.** To measure participants’ interest in science-fiction, we administered the Science-Fiction Hobbyism Scale (Laakasuo et al. 2018; translated by us). The scale contains 11 items and participants are asked to rate them on a seven-point Likert scale ranging from “1—strongly disagree” to “7—strongly agree”. A higher mean value indicated more interest in science-fiction.

**Purity Norms.** Purity norms were assessed using the Moral Foundations Questionnaire (MFQ; see Graham et al. 2009) in its German translation by Jöckel et al. (2012). This questionnaire contains 31 items covering five moral foundations. The items are rated on a six-point Likert scale ranging from either “1—not at all relevant” to “6—extremely relevant” or “1—strongly disagree” to “6—strongly agree”. For the purpose of the present research questions, we only calculated a mean across the 5-item sanctity/purity subscale.

### 2.3. Study Procedure

Participating in our study took about 60 min, separated into two survey parts (approx. 30 min each). In both parts, the participants first generated a code so that we were able to merge their data. In the first part of the survey, the participants reported their demographic data (i.e., gender, age, education, interest in technology, and German language skills). Then, they answered the first half of the enhancement vignettes (either active or passive vignettes), followed by the BFI-K personality questionnaire and the second half of the enhancement vignettes.^8^ At the end of this first survey part, the participants completed the Science-Fiction Hobbyism Scale and the MFQ. Finally, we asked participants for their email addresses so that we could send them the second survey part, which we did two days after they had finished the first one.

In the second part of the survey, the participants first answered the self-estimation questionnaires (multi-item and single-item) and then the numerical, verbal, and figural tasks of the ISA. At the end of these intelligence measures, the participants were asked whether they had used any unauthorized help. Then, they answered the Dirty-Dozen Questionnaire and the HSNS Scale. Finally, participants answered the ITIS on implicit intelligence theories.

## 3. Results

We tested all research questions and hypotheses two-tailed and the common statistical assumptions were met unless otherwise stated. Exploratory analyses are highlighted respectively.

### 3.1. Exploratory Factor Analysis

For this study, we created eight enhancement vignettes that can—on a theoretical basis—be separated into passive and active enhancement methods. However, to investigate whether our theory-based assignment of methods to passive and active enhancement holds up, we conducted an exploratory factor analysis (Maximum-Likelihood method with Varimax rotation). Both Bartlett’s test of sphericity, χ2(28) = 495.65, *p* < 0.001, and the Kaiser-Meyer-Olkin value, KMO = 0.82, indicated that the collected data is adequate for computing a factor analysis. The Scree plot and Eigenvalues above the criterion of 1 suggested an extraction of two factors that together explain 56.01% of the variance in our data. Five out of eight enhancement methods were strongly loading on factor 1 (Eigenvalue = 3.26), whereas the other three were loading on a second factor (Eigenvalue = 1.22; see Table 3). Thus, based on this analysis, we grouped the eight enhancement methods into five passive (pharmacological enhancement, current-based enhancement, genetic enhancement, mind upload, and brain-machine interface) and three active enhancement methods (working memory training, game-based enhancement, neurofeedback training) for our main analyses.

### 3.2. Comparison of Enhancement Methods

To analyze the differences between the acceptance of each enhancement method, we performed two ANOVAs—one for passive and one for active enhancement methods^9^. As the assumption of sphericity was violated for both analyses, we based our interpretation on the Greenhouse-Geisser corrected test statistics.^10^ We observed a significant difference between our five passive enhancement methods, *F*(3.84, 983.14) = 15.83, *p* < 0.001, *η*_p_^2^ = 0.06. Post hoc *t*-tests suggested a higher acceptance of the pharmacological and current-based enhancement methods than the other passive enhancement methods (i.e., genetic enhancement, mind upload, brain-machine interface; all *t*s(256) ≥ 4.29, all *p*s < 0.001, all *ds* ≥ 0.27; see Table 2 for means and standard deviations). Pharmacological and current-based enhancement did not differ in their acceptance, *t*(256) = 0.48, *p* = 0.628; *d* = 0.03, and neither did the other three enhancement methods, all *t*s(256) ≤ 1.25, all *p*s ≥ 0.211; all *ds* ≤ 0.08.

For active enhancement, we also observed a significant difference between the three respective methods, *F*(1.90, 487.16) = 30.76, *p* < 0.001; *η*_p_^2^ = 0.11. Post hoc *t*-tests showed a higher acceptance for working memory training than game-based enhancement and neurofeedback training, all *t*s(256) ≥ 5.81, all *p*s < 0.001; all *d*s ≥ 0.36. Game-based enhancement and neurofeedback training did not differ in their acceptance, *t*(256) = 1.26, *p* = 0.207; *d* = 0.08. In an additional exploratory analysis, we compared the acceptance of the grouped passive and active enhancement methods with a *t*-test. We observed a higher acceptance for active than passive enhancement methods, *t*(256) = −20.85, *p* < 0.001, *d* = −1.30 (for means and standard deviations see Table 2).

### 3.3. Correlational Analyses

To investigate our preregistered research questions (RQ1 to RQ3) and hypotheses (H1 to H4), we conducted two-tailed Pearson correlations. We additionally computed Bayes factors (BF_01_) to illustrate the relative evidence for the null hypothesis as compared to the alternative hypothesis. The results are displayed in Table 4. Additionally, these results can also be found in Table 1 together with our detailed research questions/hypotheses. Our control variable age shows a negative correlation with both acceptance of passive and active enhancement methods. Furthermore, education was negatively and gender positively associated with the acceptance of passive enhancement methods (i.e., men show a higher acceptance).

With regard to RQ1, we observed a positive correlation between measured general intelligence and acceptance of active enhancement, but not passive enhancement. Self-estimated general intelligence did not correlate with the acceptance of passive or active enhancement (RQ2). Additionally, concerning RQ3, we did not observe a significant correlation between individuals’ implicit theories of intelligence and their acceptance of passive/active enhancement. We observed the expected negative correlation between conscientiousness and acceptance of passive enhancement, but unexpectedly not active enhancement (see H1). Furthermore, openness was—unexpectedly—positively related to acceptance of active enhancement. No other significant relationships between Big Five personality traits and acceptance of passive/active enhancement were observed, meaning that we could not confirm our hypotheses about negative associations between agreeableness and enhancement. With regard to the Dark Triad traits and vulnerable narcissism, we observed the expected positive correlations between Machiavellianism as well as grandiose narcissism with both acceptance of passive and active enhancement methods (see H2). Vulnerable narcissism was only positively correlated to the acceptance of passive enhancement, and, unexpectedly, psychopathy was not related to either form of acceptance. Confirming our H3, we observed a positive correlation between science-fiction hobbyism and the acceptance of passive and active enhancement methods. Purity norms were negatively correlated to the acceptance of active enhancement, but not to the acceptance of passive enhancement, only partly supporting H4.

Correlations between the intelligence sub-facets and the acceptance of passive and active enhancement methods can be found in the Supplementary Materials (see Table S1, https://osf.io/du39z/). Furthermore, intercorrelations of intelligence and personality measures are depicted in Table S2 of the supplement.

### 3.4. Hierarchical Regression Analyses

To examine which factors together contribute to the prediction of the acceptance of passive/active enhancement (see RQ4), we originally planned hierarchical regression analyses in four steps (see https://osf.io/urwxt for details). Different from our original plan, the hierarchical regression analysis with criterion “acceptance of passive enhancement” only contained three steps, as we did not observe significant correlations with measured and self-estimated intelligence as well as the implicit theories of intelligence (i.e., step 2 of our preregistered analyses plan was skipped). Nevertheless, as a first step, we entered the control variables age, education, and gender. Together, they predicted 9% of the variance in the acceptance of passive enhancement. As the second step, we additionally included conscientiousness and Machiavellianism as well as grandiose and vulnerable narcissism, which explained an additional 5% of the variance. Finally, we included science-fiction hobbyism, which additionally predicted 4% of the variance. Thus, a total of 18% of the variance of the acceptance of passive enhancement was explained by those predictors (for details see Table 5).

For the criterion “acceptance of active enhancement”, we first entered the control variable age, which explained 12% of the variance. Then, we entered general intelligence but it did not explain incremental variance in acceptance. As the third step, we entered openness, Machiavellianism, and grandiose narcissism, which together explained an additional 7% of the variance. In our final model, we added science-fiction hobbyism and purity norms with 3% of incrementally explained variance. Altogether, the included predictors explained 22% of the variance in the acceptance of active enhancement methods.^11^

As the acceptance of active enhancement methods was significantly correlated to self-estimated verbal intelligence (single- and multi-item measures; see Table S1 in the supplement), we conducted an additional exploratory hierarchical regression analysis including these predictors. This analysis can be found in Table S3 of the supplement. The results are in line with our main analysis depicted in Table 5. Thus, after accounting for all factors, only age, openness, and science-fiction hobbyism predicted the acceptance of active enhancement.

## 4. Discussion

In the present study, we investigated individual differences in the acceptance of passive and active cognitive-enhancement methods and their correlation with intelligence and personality traits. First, in exploratory factor analysis, we observed that the eight tested enhancement methods can be grouped into five passive (pharmacological enhancement, current-based enhancement, genetic enhancement, mind upload, and brain-machine interface) and three active enhancement methods (working memory training, game-based enhancement, neurofeedback training). Within the passive enhancement methods, acceptance was higher for pharmacological and current-based enhancement than for the other methods. Furthermore, working memory training entailed a higher acceptance than the other two active enhancement methods. Overall, acceptance was higher for active than passive enhancement methods.

As our main research questions, we tested the relationships of both psychometrically measured and self-estimated intelligence with the acceptance of passive and active enhancement methods (RQ1 & RQ2; for a more detailed presentation of the research questions and hypotheses see Table 1). If we observed significant correlations, we additionally investigated the incremental variance of intelligence measures beyond control variables in the acceptance of passive/active enhancement. While we did not observe a significant correlation between measured general intelligence and the acceptance of passive enhancement, we found a small positive correlation with active enhancement. However, in the following multiple regression model, general intelligence did not predict the acceptance of active enhancement over and above the control variable age. Self-estimated intelligence and acceptance of passive or active enhancement were not significantly related. Moreover, we did not observe a relationship between individuals’ implicit theories of intelligence and their acceptance of passive or active enhancement (RQ3).

Next, we investigated whether we can replicate previously reported associations between personality traits and acceptance of enhancement. With regard to the Big Five traits, higher conscientiousness was related to lower acceptance of passive (but not active) enhancement, partly confirming our hypotheses (see H1). However, we did not detect the expected negative associations between agreeableness and acceptance of active or passive enhancement. Instead, we found an unexpected positive association of openness with the acceptance of active enhancement. When we investigated which traits can explain variance over and above control variables and—in the case of active enhancement—general intelligence, higher conscientiousness predicted lower acceptance of passive enhancement and higher openness predicted higher acceptance of active enhancement. From the Dark Triad, both Machiavellianism and grandiose narcissism showed small positive correlations with the acceptance of both types of enhancement, confirming our respective hypotheses (see H2). For vulnerable narcissism, we observed the expected positive correlation only for acceptance of passive enhancement; for psychopathy, neither correlation reached significance. Moreover, no Dark Triad trait (including vulnerable narcissism) explained significant incremental variance of the acceptance of passive or active enhancement in the multiple regression model beyond the other included variables (e.g., control variables and personality traits; see Table 5).

Confirming our hypothesis (H3), higher science-fiction hobbyism was significantly correlated to a higher acceptance of passive and active enhancement methods. Science-fiction hobbyism was also shown to be the strongest predictor for passive enhancement (*β* = 0.22) over and above included control variables and personality traits. For active enhancement, science-fiction hobbyism was also a significant predictor (in addition to the other included variables; see Table 5), but to a lower extent (*β* = 0.15). With regard to our last preregistered hypothesis (H4), we observed that higher purity norms were significantly correlated to lower acceptance of active but not passive enhancement. However, in the multiple regression model, purity norms were not a significant predictor of the acceptance of active enhancement over and above control variables, intelligence, personality traits, and science-fiction hobbyism.

Notably, single control variables showed associations with the acceptance of enhancement. Age predicted both the acceptance of passive and active enhancement, suggesting that younger individuals are more willing to enhance their cognitive abilities. For active enhancement, age was the strongest predictor of acceptance (*β* = −0.29). Gender significantly contributed to the model explaining acceptance of passive enhancement (men showing higher acceptance)—but only until science-fiction hobbyism was entered into the equation.

To summarize, in the final model, the acceptance of passive enhancement was negatively predicted by age and conscientiousness as well as positively predicted by science-fiction hobbyism, with an overall explained variance of 18%. The acceptance of active enhancement was negatively predicted by age as well as positively predicted by openness and science-fiction hobbyism, with an overall explained variance of 22%. We discuss these findings in the following sections.

### 4.1. Structure of Acceptance of Cognitive Enhancement Methods

From a theoretical point of view, we expected that our eight cognitive enhancement methods would equally cluster into two factors—one passive and one active enhancement factor with four methods each. Our data indeed confirmed this hypothesized categorization for seven of the enhancement methods, but unexpectedly the brain-machine interface method was assigned to passive instead of active enhancement. We believe this might be due to the passive part of this scenario (i.e., going under surgery to get a brain implant) being much more central in the vignette than the active part (i.e., performing training after receiving the implant; for the full vignettes see https://osf.io/du39z/). Thus, participants might view brain-machine interfaces as a rather passive form of cognitive enhancement. In contrast, the three active enhancement methods (working memory training, game-based enhancement, and neurofeedback training) did not include any passive part, making active training the central component of the vignette. Another difference between the passive and active enhancement methods was their invasiveness. While all enhancement methods primarily loading onto the passive enhancement factor (including brain-machine interfaces) included the intake of substances or the modification of one’s physiology, all methods loading onto the active enhancement factor were non-invasive.

### 4.2. Comparison of Cognitive-Enhancement Methods

Within the group of passive enhancement methods, pharmacological and current-based enhancement seem more accepted than the other methods (for a similar finding, see Schönthaler et al. 2022). One reason for this could be the higher realizability of these two methods. While smart drugs and brain-stimulation techniques are already available for most individuals, gene editing, mind upload, and brain-machine interfaces are more futuristic enhancement methods and not available to the general public (or not even developed yet). Another reason might be that both the procedures and consequences of pharmacological and current-based enhancement might seem less extreme than those of the other three methods. For instance, while smart drugs entail the simple intake of a drug that might only induce short-term effects on cognitive performance (presumably at least when not used/applied for longer times), gene editing entails a sophisticated medical procedure that likely is irreversible and has life-long effects.

For active enhancement, working memory training had a higher acceptance than game-based enhancement and neurofeedback training. People might be more familiar with working memory training due to the availability of many smartphone apps that supposedly help to improve one’s memory, thus, resulting in a higher acceptance of this method. Doing this type of working memory training is also easier than, for instance, visiting a neurofeedback training center.

When exploratorily comparing the grouped passive and active enhancement methods, we observed a higher acceptance of active than passive enhancement. This might be due to the less futuristic and more realistic nature of active in comparison to the passive enhancement methods. Passive enhancement includes methods such as gene editing and mind upload, which might be hard to picture for the general population. Thus, people might be more drawn towards already broadly available methods, such as working memory training, gaming, and neurofeedback training. In addition, the invasiveness of passive compared to active enhancement methods might decrease acceptance. In line with our findings, Koverola et al. (2022) observed that people show a (numerically) greater approval of enhancement when it is aiming at optimal performance (i.e., restoring optimal abilities one had during youth) than at superhuman performance (i.e., unlimited cognitive abilities). Thus, individuals might be more reluctant towards methods that aim at superhuman abilities—such as mind upload in our study (i.e., aiming at digital immortality).

### 4.3. Predictors of Passive and Active Enhancement

#### 4.3.1. Intelligence

One goal of our study was to test whether psychometrically measured and self-estimated general intelligence predicted the acceptance of passive and active enhancement methods. We introduced two competing hypotheses—the rich-get-richer and compensation hypotheses. While the rich-get-richer hypothesis suggests a positive relationship between measured and/or self-estimated intelligence and acceptance of enhancement (i.e., more acceptance with higher intelligence), the compensation hypothesis suggests a negative relationship (i.e., more acceptance with lower intelligence). In our study, we did not find support for either of these hypotheses. While there was a small positive correlation between measured intelligence and the acceptance of active enhancement (supporting the rich-get-richer hypothesis), this relationship diminished in our multiple regression model (i.e., after controlling for age). Thus, measured and self-estimated intelligence do not seem to account for individuals’ willingness to enhance their cognitive abilities.^12^

Based on the theoretical background, we focused on a linear relationship between intelligence measures and the acceptance of enhancement in our study. However, it might be the case that both the rich-get-richer and compensation hypotheses actually apply at the same time, which would mean that both people of rather high and low intelligence are willing to get enhanced (i.e., non-linear associations might be observed). While more intelligent people might want to enhance themselves to become even smarter, less intelligent people might want to enhance themselves as a compensation strategy. To empirically test this assumption, follow-up studies might focus on testing, for instance, quadratic relationships between intelligence measures and the acceptance of enhancement as well as on individuals’ motives to enhance themselves.

Furthermore, it is mostly unclear whether the acceptance of enhancement (and enhancement behavior itself) is influenced primarily by a person’s internal factors, the given situation, or both. On one hand, following a dispositionalist view on enhancement, its acceptance might be predicted by internal factors such as intelligence or personality traits. On the other hand, the acceptance of enhancement (and enhancement behavior itself) might depend on the given situation as suggested by a situationist view. In addition to those two possibilities (the dispositionalist and situationist view), internal factors and a situation might interact when it comes to the acceptance of enhancement (an interactionist view). Thus, measured/self-estimated intelligence might only act as a predictor of enhancement in certain situations. For example, attaining academic achievements might be a common motivation for using pharmacological enhancement (see Daubner et al. 2021). If individuals experience situations with performance pressure, individual differences with regard to enhancement might emerge as certain individuals (e.g., less intelligent individuals) might be more willing to get enhanced (for a first indication of this, see Bard et al. 2018). In our study, however, the participants were asked to read rather general enhancement vignettes without inducing performance pressure, which might have made enhancement less attractive. It might, thus, be a promising approach for future studies to test the acceptance of enhancement (and predictors thereof) in different situations.

As individuals are oftentimes not able to correctly estimate their intelligence, their decisions and, thus, behaviors might be based on false metacognitive beliefs (see, for example, Hofer et al. 2022). Thus, we were interested to test whether self-estimated intelligence rather than psychometrically measured intelligence predicts the acceptance of enhancement. However, this does not seem to be the case. Neither metacognitive beliefs about one’s own intelligence nor measured intelligence contribute to the acceptance of enhancement and potentially enhancement behavior itself—at least as suggested by this first study. Similar to measured and self-estimated intelligence, participants’ implicit theories about intelligence (i.e., whether intelligence is a fixed or malleable trait) were not related to the acceptance of passive and active enhancement. Thus, higher beliefs towards the incremental theory, which suggests that intelligence can be changed, are not related to a higher willingness to get enhanced. It must be noted, that while our enhancement vignettes described the enhancement of one’s *cognitive abilities*, the questionnaire on implicit theories of intelligence specifically focused on *intelligence*. This discrepancy could be a reason why implicit theories of intelligence were not related to the acceptance of cognitive enhancement. Nevertheless, in line with our findings, another study by Champagne et al. (2019) observed no relationship between entity beliefs and attitudes toward smart drugs. Thus, potentially implicit theories of intelligence do not account for the willingness to get enhanced.

#### 4.3.2. Personality

In previous research, certain Big Five traits were only shown to be rather weak predictors of assumptions about and the acceptance of different cognitive enhancement methods (e.g., Grinschgl et al. 2022; Schönthaler et al. 2022). Furthermore, the observed relationships were rather inconsistent. Nevertheless, based on a previous study by Schönthaler et al. (2022), we expected a negative relationship between agreeableness as well as conscientiousness and the acceptance of passive/active enhancement methods. We expected no significant associations for extraversion, openness, and neuroticism. We partly confirmed these hypotheses. A higher conscientiousness predicted lower acceptance of passive enhancement. This is in line with findings showing that conscientious individuals try to avoid behaviors that might risk their health (e.g., Hampson et al. 2007) and that they likely follow group norms (Hogan and Ones 1997). Additionally, individuals higher in conscientiousness might be more willing to put effort into their performance gains and, thus, are reluctant to use passive cognitive enhancement. Furthermore, conscientious individuals might not see enhancement as a fair way to improve performance and/or cognition. Conscientious individuals tend to follow intrinsic motives (e.g., personal standards, self-oriented perfectionism) when performing tasks (Smith et al. 2019; Stoeber et al. 2009), which might lead to enhancement not being an attractive method to increase cognition for them (cf. Stoeber and Hotham 2016). While these assumptions are in line with the observed negative relationship between conscientiousness and the acceptance of passive enhancement, conscientiousness was not related to active enhancement. Thus, this personality trait does not seem to play a (large) role when it comes to actively putting effort into increasing one’s cognitive abilities.

We further observed that a higher openness predicted more acceptance of active enhancement. Thus, more open individuals might be more willing to put effort into enhancing their cognition. Moreover, more open individuals might show a higher willingness to try new technologies and might strive for new experiences and challenges (see Matthews et al. 2021). In line with the study by Schönthaler et al. (2022), openness was not related to the acceptance of passive enhancement methods (see also Laakasuo et al. 2018). This assumption is also supported in a study by Grinschgl et al. (2022), which showed that more open individuals derived more negative consequences accompanying passive enhancement methods and might, thus, not be particularly willing to get enhanced.

We did not observe significant relationships between acceptance of enhancement and the remaining Big Five traits, namely agreeableness, extraversion, and neuroticism (which is also supported by rather high Bayes Factors in support of the null hypothesis). The lack of associations of acceptance with extraversion and neuroticism is in line with past findings (Laakasuo et al. 2018; Schönthaler et al. 2022). It also mostly conforms with no significant associations (Laakasuo et al. 2018) or mixed findings depending on the specific enhancement method reported for agreeableness (Schönthaler et al. 2022). Potentially, findings might differ when participants have to assess their willingness to get enhanced in a stressful situation. For instance, Myrseth et al. (2018) observed a negative relationship between extraversion and the use of pharmacological enhancement with the goal to ease one’s nerves in performance-challenging situations. Thus, varying the context of applying cognitive enhancement might again provide more differentiated insights into the relationship between personality and acceptance of enhancement (see also Schönthaler et al. 2022).

Furthermore, higher Machiavellianism and grandiose narcissism were related to higher acceptance of both passive and active enhancement. Additionally, higher vulnerable narcissism was related to higher acceptance of passive enhancement—but all associations can be considered as small. Replicating the findings of Schönthaler et al. (2022), these traits did not predict the acceptance of passive and active enhancement methods when accounting for control variables and the Big Five traits. Thus, higher Machiavellian, psychopathic, and grandiose as well as vulnerable narcissistic tendencies seem not to support the willingness to enhance one’s cognitive abilities—at least not to a large degree. This finding stands in contrast with a study by Laakasuo et al. (2021) who observed Machiavellianism as a strong predictor of the acceptance of mind upload—but no personality traits beyond the Dark Triad were included in this study (see also Mayor et al. 2020 for a related finding). Nevertheless, potentially Dark Triad traits only account for the acceptance of cognitive enhancement when it comes to specific enhancement methods.

#### 4.3.3. Further Predictors (Age, Science-Fiction Hobbyism, Purity Norms)

Beyond personality traits, participants’ age, and science-fiction hobbyism predicted both acceptance of passive and active enhancement. Lower age and higher interest in science-fiction were related to higher acceptance of enhancement. Thus, younger individuals might be more open to the transhumanistic idea of enhancement. Furthermore, younger individuals might be under more performance pressure during their education and/or at the beginning of their careers, making cognitive enhancement potentially more attractive. As hypothesized and also observed in previous studies (Koverola et al. 2022; Laakasuo et al. 2018), the preference to consume science-fiction literature, movies, and series is accompanied by a higher willingness to get enhanced. This might be due to (futuristic) enhancement methods being more familiar to those individuals.

While purity norms were related to participants’ acceptance of active (but not passive) enhancement, they did not account for incremental variance in the multiple regression model over and the above the other included variables (see Table 5). Thus, in our study, the hypothesized association between purity norms which includes values such as pureness of one’s body, naturalness, and decency, and the acceptance of enhancement could not be supported. In contrast, Laakasuo et al. (2018) showed a negative association between purity norms and approval of mind upload—a method that especially affects one’s body. However, no further enhancement methods were tested in that study. Purity norms might–if at all–only account for the acceptance of cognitive enhancement when it comes to single enhancement methods.

## 5. Limitations and Future Directions

We need to highlight some limitations of our study as well as future directions for research that arise from those: First, it needs to be considered that we administered the intelligence tasks online (i.e., participants performed the study at home or anywhere else). Although we asked our participants to strictly follow the task instructions and to not use any external help, we cannot rule out the possibility that participants might have cheated during task performance or been distracted by surrounding factors. Therefore, we aim at replicating this study in a laboratory setting. Second, our tested sample includes rather young, well-educated individuals. Thus, our sample is not highly diverse which might also be accompanied by relatively uniform opinions about enhancement. Notably, standard deviations on the acceptance of enhancement in our study do not appear to be too small. Nevertheless, for further individual differences studies, we argue to include more diverse samples in order to obtain a more representative view of cognitive enhancement. Additionally, it might be interesting to collect data from participants from different cultural backgrounds and under different circumstances (e.g., enhancement in performance-challenging situations). Finally, we argue for testing additional factors that might account for individual differences in the acceptance of human enhancement. For instance, as science-fiction hobbyism seems to be a stable (and relatively strong) predictor of the acceptance of enhancement, other interests (e.g., professional interests) might also act as predictors. Hence, testing the RIASEC interests (Holland 1997) as predictors of the acceptance of enhancement might be a promising approach for future research.

## 6. Conclusions

The present study was the first attempt to test a broad range of predictors (intelligence measures, personality traits, specific interests, and values) for the acceptance of cognitive enhancement. We included a set of different cognitive enhancement methods that could be separated into passive and active enhancement methods. Unexpectedly, intelligence measures did not predict the acceptance of passive or active enhancement over and above control variables. Instead, for the acceptance of passive enhancement, we observed lower age, lower conscientiousness, and higher science-fiction hobbyism as predictors. Similarly, lower age, higher openness, and higher science-fiction hobbyism predicted higher acceptance of active enhancement. Thus, while only selected personality traits seem to play a role in the acceptance of enhancement (see also Schönthaler et al. 2022), individuals’ age and science-fiction hobbyism currently seem to be the most consistent and strongest predictors of acceptance of passive and active enhancement. Overall, the variance in the acceptance of enhancement explained in our final model, including all correlated control variables, intelligence, personality traits, and interests/norms, can be considered moderate—leaving room for more potential predictors to be tested in future studies. We further argue that future work in this area would benefit from more representative samples. Cognitive enhancement is an emerging topic both in science and the public. Thus, individual differences with regard to enhancement should be extensively researched to, for instance, reduce the side effects of enhancement and/or provide support to those applying it.

## Figures and Tables

**Table 1:** Correlative Research Questions, Hypotheses, and Results.

RQ/H	Variables		*r*_hyp_	*r*_result_
	**Intelligence**			
RQ1.a	Measured Intelligence	Acceptance of active enhancement		**0.14**
RQ1.p		Acceptance of passive enhancement		−0.01
RQ2.a	Self-estimated Intelligence	Acceptance of active enhancement		0.05
RQ2.p		Acceptance of passive enhancement		−0.07
RQ3.a	Implicit Theories of Intelligence	Acceptance of active enhancement		−0.07
RQ3.p		Acceptance of passive enhancement		0.03
	**Big Five Traits**			
H1.1.a	Agreeableness	Acceptance of active enhancement	−	0.04
H1.1.p		Acceptance of passive enhancement	−	−0.02
H1.2.a	Conscientiousness	Acceptance of active enhancement	−	<0.01
H1.2.p		Acceptance of passive enhancement	−	**−0.22**
H1.3.a	Extraversion	Acceptance of active enhancement	0	0.11
H1.3.p		Acceptance of passive enhancement	0	−0.06
H1.4.a	Openness	Acceptance of active enhancement	0	**0.24**
H1.4.p		Acceptance of passive enhancement	0	0.07
H1.4.a	Neuroticism	Acceptance of active enhancement	0	−0.01
H1.4.p		Acceptance of passive enhancement	0	0.14
	**Dark Triad Traits** (incl. vulnerable narcissism)			
H2.1.a	Machiavellianism	Acceptance of active enhancement	+	**0.13**
H2.1.p		Acceptance of passive enhancement	+	**0.14**
H2.2.a	Psychopathy	Acceptance of active enhancement	+	−0.01
H2.2.p		Acceptance of passive enhancement	+	0.02
H2.3.a	Grandiose Narcissism	Acceptance of active enhancement	+	**0.19**
H2.3.p		Acceptance of passive enhancement	+	**0.13**
H2.4.a	Vulnerable Narcissism	Acceptance of active enhancement	+	0.04
H2.4.p		Acceptance of passive enhancement	+	**0.15**
	**Interests and Values**			
H3.a	Science-fiction Hobbyism	Acceptance of active enhancement	+	**0.24**
H3.p		Acceptance of passive enhancement	+	**0.27**
H4.a	Purity Norms	Acceptance of active enhancement	−	**−0.17**
H4.p		Acceptance of passive enhancement	−	−0.09

*Note*. H = Hypothesis. RQ = Research Question. *r*_hyp_ = Hypothesized correlation. *r*_result_ = Correlation result. + = Positive Correlation. − = Negative Correlation. 0 = Correlation of about zero. For RQ1.a to RQ3.p, we had no hypotheses. Significant correlations in bold (*p* < 0.05). RQ4.a and RQ4.p (multiple regression; not included) concerned the amount of variance in the acceptance of active/passive enhancements explained by intelligence, Big Five traits, Dark Triad traits, and interests and values.

**Table 2:** Descriptive Statistics of All Variables.

Variable	*M*	*SD*	Skewness	Kurtosis	Cronbach’s *α*
**Acceptance of Enhancement**					
Passive Enhancement Methods	2.85	1.05	0.34	−0.01	0.75
Pharmacological Enhancement	3.19	1.49	0.1	−0.89	-
Current-based Enhancement	3.14	1.39	0.16	−0.73	-
Genetic Enhancement	2.72	1.46	0.48	−0.66	-
Mind Upload	2.61	1.58	0.69	−0.67	-
Brain-Machine Interface	2.60	1.48	0.62	−0.63	-
Active Enhancement Methods	4.27	1.04	0.61	0.65	0.71
Working Memory Training	4.66	1.18	−0.85	0.71	-
Game-based Enhancement	4.14	1.44	−0.51	−0.42	-
Neurofeedback Training	4.02	1.31	−0.4	−0.63	-
**Measured Intelligence**					
Numerical Intelligence	11.19	4.42	−0.12	−0.47	0.89
Verbal Intelligence	14.80	4.09	−1.07	0.68	0.84
Spatial Intelligence	9.17	3.73	0.04	−0.66	0.76
General Intelligence Score (z-score)	0	0.79	−0.48	−0.11	0.71
**Self-Estimated Intelligence**					
Single Item (IQ)					
General Intelligence	108.33	11.31	−0.25	0.33	-
Numerical Intelligence	103.30	15.14	−0.81	<0.01	-
Verbal Intelligence	108.04	12.54	−0.19	0.69	-
Spatial Intelligence	102.93	13.8	−0.46	0.78	-
Multi-Item (Questionnaire)					
Numerical	3.18	0.95	−0.25	−0.64	0.95
Verbal	3.53	0.68	−0.09	−0.31	0.87
Spatial	3.26	0.82	−0.29	−0.62	0.89
**Implicit Theories of Intelligence**	3.49	0.88	−0.05	0.01	0.91
**Big Five**					
Agreeableness	3.19	0.83	−0.23	−0.48	0.67
Conscientiousness	3.69	0.77	−0.45	−0.34	0.72
Extraversion	3.47	0.96	−0.34	−0.55	0.85
Openness	4.03	0.65	−0.67	−0.03	0.66
Neuroticism	3.08	1.04	0.09	−1.04	0.83
**Dark Triad**					
Machiavellianism	3.02	1.49	0.84	0.4	0.77
Psychopathy	2.82	1.47	1.05	0.86	0.62
Grandiose Narcissism	4.18	1.69	−0.02	−0.79	0.82
**Vulnerable Narcissism**	2.85	0.6	0.03	−0.09	0.74
**Science-fiction Hobbyism**	2.94	1.03	0.41	−0.27	0.85
**Purity Norms**	2.84	0.93	0.27	−0.34	0.70

*Note*. *N* = 257. A skewness or kurtosis above 1 suggests non-normality–which is the case for verbal intelligence, neuroticism, and psychopathy.

**Table 3:** Rotated factor matrix.

	Factor 1 (Passive Enhancement)	Factor 2 (Active Enhancement)
Pharmacological Enhancement	**0.57**	0.21
Current-based Enhancement	**0.47**	0.35
Genetic Enhancement	**0.71**	0.17
Mind Upload	**0.56**	0.09
Brain-machine Interface	**0.57**	0.09
Working Memory Training	0.15	**0.68**
Game-based Enhancement	0.26	**0.52**
Neurofeedback Training	0.19	**0.74**

*Note*. *N* = 257. Extraction method: Maximum Likelihood. Rotation method: Varimax. Factor loadings above 0.40 are depicted in bold.

**Table 4:** Correlational Analyses of Main Variables.

	Passive Enhancement		Active Enhancement	
	*r* [95% CI]	*BF*_01_	*r* [95% CI]	*BF*_01_
**Control Variables**				
Age	−0.20 *** [−0.30; −0.09]	0.10	−0.35 *** [−0.46; −0.20]	<0.01
Education	−0.13 * [−0.25; −0.01]	1.65	−0.02 [−0.15; 0.09]	20.10
Gender	0.15 * [0.02; 0.27]	1.00	−0.01 [−0.16; 0.12]	19.58
**Measured General Intelligence (z-score)**	−0.01 [−0.13; 0.12]	20.03	0.14 * [0.02; 0.26]	1.76
**Self-estimated General Intelligence (IQ)**	−0.07 [−0.20; 0.09]	11.59	0.05 [−0.08; 0.19]	13.68
**Implicit Theories of Intelligence**	0.03 [−0.10; 0.16]	17.19	−0.07 [−0.19; 0.06]	11.05
**Big Five**				
Agreeableness	−0.02 [−0.14; 0.10]	18.95	0.04 [−0.08; 0.15]	16.89
Conscientiousness	−0.22 *** [−0.33; −0.09]	0.04	<0.01 [−0.12; 0.12]	20.15
Extraversion	−0.06 [−0.18; 0.07]	12.89	0.11 [−0.01; 0.23]	4.33
Openness	0.07 [−0.05; 0.19]	10.13	0.24 *** [0.12; 0.36]	0.01
Neuroticism	0.14 * [−0.005; 0.28]	1.46	−0.01 [−0.14; 0.12]	19.76
**Dark Triad**				
Machiavellianism	0.14 * [0.01; 0.26]	1.73	0.13 * [0.01; 0.25]	2.02
Psychopathy	0.02 [−0.11; 0.16]	18.77	−0.01 [−0.17; 0.13]	19.60
Grandiose Narcissism	0.13 * [0.02; 0.25]	1.93	0.19 ** [0.32; 0.56]	0.20
**Vulnerable Narcissism**	0.15 * [0.02; 0.28]	1.07	0.04 [−0.06; 0.16]	15.60
**Science-fiction Hobbyism**	0.27 *** [0.15; 0.38]	<0.01	0.24 *** [0.12; 0.36]	0.01
**Purity Norms**	−0.09 [−0.22; 0.05]	7.72	−0.17 ** [−0.29; −0.04]	0.42

*Note*. * *p* < 0.05. ** *p* < 0.01. *** *p* < 0.001. *N* = 257. *BF*_01_ indicates evidence for the null hypothesis over the alternative hypothesis. Education was measured with a 7-point ordinal scale, thus we calculated Spearman correlations for this variable. For gender, a value of 1 indicates females and 2 males. For non-normally distributed variables (neuroticism and psychopathy; see Table 1), interpretation is based on 95% BCa bootstrapping confidence intervals for 2000 samples (depicted in brackets for all variables).

**Table 5:** Multiple Hierarchical Regression Analyses with the Criteria “Acceptance of Passive Enhancement” and “Acceptance of Active Enhancement”.

		*R*^2^	∆*R*^2^	∆*F*	*β*	*t*
**Passive Enhancement**						
Model 1		0.09	0.09	8.02 ***		
	Age				−0.21	−3.39 **
	Education				−0.12	−2.05 *
	Gender				0.18	3.01 **
Model 2		0.13	0.05	3.27 *		
	Age				−0.17	−2.78 **
	Education				−0.10	−1.64
	Gender				0.15	2.45 *
	Conscientiousness				−0.15	−2.37 *
	Machiavellianism				0.03	0.49
	Grandiose Narcissism				−0.07	1.02
	Vulnerable Narcissism				0.10	1.59
Model 3		0.18	0.04	13.39 ***		
	Age				−0.14	−2.29 *
	Education				−0.12	−1.96
	Gender				0.11	1.81
	Conscientiousness				−0.13	−2.13 *
	Machiavellianism				0.05	0.70
	Grandiose Narcissism				0.07	1.05
	Vulnerable Narcissism				0.07	1.21
	Science-fiction Hobbyism				0.22	3.66 ***
**Active Enhancement**						
Model 1		0.12	0.12	34.79 ***		
	Age				−0.35	−5.89 ***
Model 2		0.12	<0.01	0.82		
	Age				−0.33	−5.48 ***
	General Intelligence (z-score)				0.05	0.91
Model 3		0.19	0.07	7.43 ***		
	Age				−0.32	−5.39 ***
	General Intelligence (z-score)				0.01	0.25
	Openness				0.22	3.77 ***
	Machiavellianism				0.09	1.44
	Grandiose Narcissism				0.09	1.46
Model 4		0.22	0.03	4.71 *		
	Age				−0.29	−5.03 ***
	General Intelligence (z-score)				−0.04	−0.58
	Openness				0.18	3.19 **
	Machiavellianism				0.08	1.33
	Grandiose Narcissism				0.09	1.50
	Science-fiction Hobbyism				0.15	2.59 *
	Purity Norms				−0.09	−1.59

*Note*. * *p* < 0.05, ** *p* < 0.01, *** *p* < 0.001; *N* = 257; For gender, a value of 1 indicates females and 2 males.

## Data Availability

Supplementary Material, data, and the analysis script are available at the Open Science Framework (https://osf.io/du39z/).

## Notes

1 Cognitive enhancement is sometimes also referred to as *neuroenhancement* (Viertbauer and Kögerler 2019).

2 https://www.neurotwin.eu/.

3 Please note that this enhancement method was later categorized as a passive enhancement method based on exploratory factor analysis. Details will be discussed in the remaining sections.

4 Please note that we are summarizing our research questions and hypotheses here. A detailed list of all research questions and hypotheses can be found in Table 1.

5 We decided to set this upper age limit as we administered intelligence tests and cognitive abilities typically decline with age.

6 By full data sets, we mean that participants fully completed both survey parts. See the following descriptions for what those survey parts entail.

7 For the purpose of this article, we only analyzed the first question.

8 We balanced whether participants were first presented with the active or passive enhancement vignettes. Thus, 51% of the participants were presented with the passive vignettes first and the active vignettes second, whereas the remaining 49% were presented with the reverse order.

9 We preregistered that each ANOVA will contain an independent variable with four levels (i.e., four enhancement methods). However, as the exploratory factor analysis suggested that passive enhancement has five levels and active enhancement has three levels, we performed the ANOVAs accordingly.

10 We mistakenly preregistered to test the assumption of variance homogeneity; however, for within-subjects ANOVAs, sphericity is the correct assumption to test.

11 As stated in our preregistration, we also ran this regression model with the reversed order (i.e., swapping steps 2 and 3). However, this did not affect our results and interpretations in a meaningful way.

12 As stated in the preregistration, we also looked at self-estimates in the different intelligence domains separately. In exploratory analyses, self-estimated verbal abilities were positively related to the acceptance of active enhancement. However, there was no significant prediction in the multiple regression model over and above the control variable age (see Table S3 in the Supplementary Materials, https://osf.io/du39z/).

## Supplementary Materials

The following supporting information can be downloaded at: https://www.mdpi.com/article/10.3390/jintelligence11060109/s1; Table S1: Correlations of acceptance of enhancement and intelligence sub-facets; Table S2: Intercorrelations of intelligence measures, personality traits, and additional variables; Table S3: Multiple hierarchical regression analysis with the criterion “acceptance of active enhancement”.
