# Correction: Grinschgl et al. (2023). Who Wants to Enhance Their Cognitive Abilities? Potential Predictors of the Acceptance of Cognitive Enhancement. *Journal of Intelligence* 11: 109

**DOI:** 10.3390/jintelligence12100092

**Published:** 2024-09-24

**Authors:** Sandra Grinschgl, Anna-Lena Berdnik, Elisabeth Stehling, Gabriela Hofer, Aljoscha C. Neubauer

**Affiliations:** 1 Institute of Psychology, University of Graz, Universitätsplatz 2, 8020 Graz, Austria; 2 Institute of Psychology, University of Bern, Fabrikstrasse 8, 3012 Bern, Switzerland

There were errors in the original publication Grinschgl et al. (2023), which are corrected here and in the original publication.

**Additional Affiliation**

There was an error regarding the affiliation 1, the correct one should be: Institute of Psychology, University of Graz, Universitätsplatz 2, 8020 Graz, Austria. There was an update regarding the affiliations for Sandra Grinschgl. In addition to affiliation 1, the additional affiliation 2 should include: Institute of Psychology, University of Bern, Fabrikstrasse 8, 3012 Bern, Switzerland. 

**Text Correction**

In Section 3.2, the degrees of freedom for the Greenhouse–Geisser corrected ANOVAs were wrongly reported. The following are the two corrected sentences:

“We observed a significant difference between our five passive enhancement methods, *F*(3.84, 983.14) = 15.83, *p* < 0.001, *η*_p_^2^ = 0.06.”

“For active enhancement, we also observed a significant difference between the three respective methods, *F*(1.90, 487.16) = 30.76, *p* < 0.001; *η*_p_^2^ = 0.11.”

Furthermore, the third paragraph in the Discussion (Section 4) included an error:

The sentence “With regard to the Big Five traits, higher conscientiousness was related to higher acceptance of passive (but not active) enhancement, partly confirming our hypotheses (see H1).” was corrected to

“With regard to the Big Five traits, higher conscientiousness was related to lower acceptance of passive (but not active) enhancement, partly confirming our hypotheses (see H1).” 

The negative relationship between conscientiousness and acceptance of passive enhancement was correctly phrased in the rest of the article. 

**Errors in Tables**

In the original publication, there were four wrongly reported Bayes factors (BF_01_) in Table 4. The Bayes factor for the correlation between conscientiousness and acceptance of active enhancement should be 20.15 (instead of <0.00), for openness and active enhancement it should be 0.01 (instead of 2.62), for psychopathy and passive enhancement it should be 18.77 (instead of 18.79), and for science-fiction hobbyism and passive enhancement it should be < 0.01 (instead of < 0.00). The corrected Table 4 appears on the next page.

There were also errors in Table 5 as published. The *F*-value for Model 3 of passive enhancement is missing *** to indicate significance (*p* < 0.001). Furthermore, in Model 3 of passive enhancement, the *t*-value for conscientiousness was reported incorrectly. Instead of a *t*-value of −0.13, the correct *t*-value is −2.13. Lastly, the t-value for age in Model 3 of active enhancement was also wrong. It should be −5.39 (instead of −5.34). The corrected Table 5 appears on the next page. 

Please note that all errors were minor (but unfortunate) typos. No further changes are required to the document. The authors state that the scientific conclusions are unaffected. None of the interpretations change. The authors apologize for any potential inconvenience caused.

This correction was approved by the Academic Editor. The original publication has also been updated. 

**Table 5:** Multiple Hierarchical Regression Analyses with the Criteria “Acceptance of Passive Enhancement” and “Acceptance of Active Enhancement”.

		*R* ^2^	∆*R*^2^	∆*F*	*β*	*t*
Passive Enhancement						
Model 1		0.09	0.09	8.02 ***		
	Age				−0.21	−3.39 **
	Education				−0.12	−2.05 *
	Gender				0.18	3.01 **
Model 2		0.13	0.05	3.27 *		
	Age				−0.17	−2.78 **
	Education				−0.10	−1.64
	Gender				0.15	2.45 *
	Conscientiousness				−0.15	−2.37 *
	Machiavellianism				0.03	0.49
	Grandiose Narcissism				−0.07	1.02
	Vulnerable Narcissism				0.10	1.59
Model 3		0.18	0.04	13.39 ***		
	Age				−0.14	−2.29 *
	Education				−0.12	−1.96
	Gender				0.11	1.81
	Conscientiousness				−0.13	−2.13 *
	Machiavellianism				0.05	0.70
	Grandiose Narcissism				0.07	1.05
	Vulnerable Narcissism				0.07	1.21
	Science-fiction Hobbyism				0.22	3.66 ***
**Active Enhancement**						
Model 1		0.12	0.12	34.79 ***		
	Age				−0.35	−5.89 ***
Model 2		0.12	<0.01	0.82		
	Age				−0.33	−5.48 ***
	General Intelligence (z-score)				0.05	0.91
Model 3		0.19	0.07	7.43 ***		
	Age				−0.32	−5.39 ***
	General Intelligence (z-score)				0.01	0.25
	Openness				0.22	3.77 ***
	Machiavellianism				0.09	1.44
	Grandiose Narcissism				0.09	1.46
Model 4		0.22	0.03	4.71 *		
	Age				−0.29	−5.03 ***
	General Intelligence (z-score)				−0.04	−0.58
	Openness				0.18	3.19 **
	Machiavellianism				0.08	1.33
	Grandiose Narcissism				0.09	1.50
	Science-fiction Hobbyism				0.15	2.59 *
	Purity Norms				−0.09	−1.59

Note. * *p* < 0.05, ** *p* < 0.01, *** *p* < 0.001; *N* = 257; For gender, a value of 1 indicates females and 2 males.

## Figures and Tables

**Table 4:** Correlational Analyses of Main Variables.

	Passive Enhancement		Active Enhancement	
	*r* [95% CI]	*BF*_01_	*r* [95% CI]	*BF*_01_
**Control Variables**				
Age	−0.20 *** [−0.30; −0.09]	0.10	−0.35 *** [−0.46; −0.20]	<0.01
Education	−0.13 * [−0.25; −0.01]	1.65	−0.02 [−0.15; 0.09]	20.10
Gender	0.15 * [0.02; 0.27]	1.00	−0.01 [−0.16; 0.12]	19.58
**Measured General Intelligence (z-score)**	−0.01 [−0.13; 0.12]	20.03	0.14 * [0.02; 0.26]	1.76
**Self-estimated General Intelligence (IQ)**	−0.07 [−0.20; 0.09]	11.59	0.05 [−0.08; 0.19]	13.68
**Implicit Theories of Intelligence**	0.03 [−0.10; 0.16]	17.19	−0.07 [−0.19; 0.06]	11.05
**Big Five**				
Agreeableness	−0.02 [−0.14; 0.10]	18.95	0.04 [−0.08; 0.15]	16.89
Conscientiousness	−0.22 *** [−0.33; −0.09]	0.04	<0.01 [−0.12; 0.12]	20.15
Extraversion	−0.06 [−0.18; 0.07]	12.89	0.11 [−0.01; 0.23]	4.33
Openness	0.07 [−0.05; 0.19]	10.13	0.24 *** [0.12; 0.36]	0.01
Neuroticism	0.14 * [−0.005; 0.28]	1.46	−0.01 [−0.14; 0.12]	19.76
**Dark Triad**				
Machiavellianism	0.14 * [0.01; 0.26]	1.73	0.13 * [0.01; 0.25]	2.02
Psychopathy	0.02 [−0.11; 0.16]	18.77	−0.01 [−0.17; 0.13]	19.60
Grandiose Narcissism	0.13 * [0.02; 0.25]	1.93	0.19 ** [0.32; 0.56]	0.20
**Vulnerable Narcissism**	0.15 * [0.02; 0.28]	1.07	0.04 [−0.06; 0.16]	15.60
**Science-fiction Hobbyism**	0.27 *** [0.15; 0.38]	<0.01	0.24 *** [0.12; 0.36]	0.01
**Purity Norms**	−0.09 [−0.22; 0.05]	7.72	−0.17 ** [−0.29; −0.04]	0.42

Note. * *p* < 0.05. ** *p* < 0.01. *** *p* < 0.001. *N* = 257. *BF*_01_ indicates evidence for the null hypothesis over the alternative hypothesis. Education was measured with a 7-point ordinal scale, thus we calculated Spearman correlations for this variable. For gender, a value of 1 indicates females and 2 males. For non-normally distributed variables (neuroticism and psychopathy; see Table 1), interpretation is based on 95% BCa bootstrapping confidence intervals for 2000 samples (depicted in brackets for all variables).
