# Supplementary material for: Who Wants to Enhance Their Cognitive Abilities? Potential Predictors of the Acceptance of Cognitive Enhancement
Source: J Intell. 2023 Jun 1;11(6):0. doi: 10.3390/jintelligence11060109 (PMC10299699; doi:10.3390/jintelligence11060109)
Supplement: Supplementary file 1 [file jintelligence-11-00109-s001.zip › jintelligence-2283736-supplementary.pdf]

Table S1

Correlations of Acceptance of Enhancement and Intelligence Sub-Facets (Exploratory Analyses)

	Passive Enhancement	Active Enhancement
	r [95% CI]	*r* [95% CI]
**Self-Estimation**		
Single-Item (IQ)		
Numerical Intelligence	-.03 [-.16; .09]	< -.01 [-.14; .13]
Verbal Intelligence	-.04 [-.18; .09]	.12* [-.01; .25]
Spatial Intelligence	-.05 [-.16; .06]	.04 [-.10; .17]
Multi-Item (Questionnaire)		
Numerical	-.07 [-.19; .06]	-.04 [-.17; .08]
Verbal	-.06 [-.19; .06]	.13* [.01; .25]
Spatial	-.08 [-.19; .04]	.01 [-.11; .13]
**Measured Intelligence**		
Numerical Intelligence	-.02 [-.14; .09]	.11 [-.01; .22]
Verbal Intelligence	-.06 [-.19; .07]	.12 [-.01; .23]
Spatial Intelligence	.06 [-.06; .18]	.11 [-.03; .23]

Note. * $p < .05$. ** $p < .01$. *** $p < .001$. $N = 257$. For non-normally distributed variables (verbal intelligence), interpretation is based on the depicted 95% BCa bootstrapping confidence intervals for 2000 samples.

Table S2

Intercorrelations of Intelligence Measures, Personality Traits, and Additional Variables (Exploratory Analyses)

	1	2	3	4	5	6	7	8	9	10	11	12	13
1) Estimated General Intelligence (IQ)	-												
2) General Intelligence (z-score)	.47***	-											
3) Implicit Theories of Intelligence	.13*	.17**	-										
4) Neuroticism	-.20***	-.08	.08	-									
5) Openness	.13*	.12	.09	.03	-								
6) Extraversion	.01	-.05	-.06	-.35***	.13*	-							
7) Agreeableness	-.12*	-.07	-.04	-.22***	.07	.31***	-						
8) Conscientiousness	-.04	-.05	-.07	-.11	.07	.25***	.07	-					
9) Machiavellianism	.01	.03	<.01	.13*	-.02	-.07	-.35***	-.13*	-				
10) Psychopathy	.19***	.05	-.04	-.12	-.14*	-.14*	-.41***	-.09	.43***	-			
11) Grandiose Narcissism	.10	.16**	.10	.19***	.07	.15*	-.16**	-.03	.44***	.20***	-		
12) Vulnerable Narcissism	-.05	-.03	.06	.54***	.05	-.45***	-.35***	-.07	.24***	.18***	.25***	-	
13) Science-Fiction Hobbyism	.18**	.19***	.02	.02	.23***	-.06	-.01	-.12	.01	-.01	.04	.09	-
14) Purity Norms	-.11	-.32***	-.11	.08	-.03	<.01	-.04	.14*	-.08	-.16**	-.10	.08	-.09

Note. * $p < .05$. ** $p < .01$. *** $p < .001$. $N = 257$.

Table S3

Multiple Hierarchical Regression Analysis with the Criterion "Acceptance of Active Enhancement" (Exploratory Analysis)

Active Enhancement						
		R^2	ΔR^2	ΔF	*β*	*t*
Model 1		.12	.12	34.79***		
	Age				-.35	-5.89***
Model 2		.14	.02	1.47		
	Age				-.33	-5.51***
	General Intelligence				.05	0.43
	Estimated Verbal Intelligence (Single-Item)				.04	0.53
	Estimated Verbal Intelligence (Multi-Item)				.08	1.13
Model 3		.20	.06	6.23***		
	Age				-.32	-5.38***
	General Intelligence (z-score)				.01	0.09
	Estimated Verbal Intelligence (Single-Item)				.03	0.35
	Estimated Verbal Intelligence (Multi-Item)				.01	0.19
	Openness				.21	3.41***
	Machiavellianism				.09	1.42
	Grandiose Narcissism				.09	1.39
Model 4		.22	.03	4.73*		
	Age				-.29	-5.02***
	General Intelligence				-.05	-0.73
	Estimated Verbal Intelligence (Single-Item)				.04	0.57
	Estimated Verbal Intelligence (Multi-Item)				<-.01	-.03
	Openness				.18	2.91**
	Machiavellianism				.09	1.35
	Grandiose Narcissism				.09	1.42
	Science-Fiction Hobbyism				.15	2.59*
	Purity Norms				-.09	-1.59

Note. *$p < .05$, **$p < .01$, ***$p < .001$; $N = 257$. Please note that entering the two self-estimate measures in separate steps does not change the results in a meaningful way.
